# Comprehensive performance analysis of an electric vehicle using multi-mode Indian drive cycles

**DOI:** 10.1038/s41598-025-02238-x

**Published:** 2025-05-21

**Authors:** Jayakara Babu Kondru, Y. P. Obulesu

**Affiliations:** School of Electrical Engineering, Vellore Institute of Technology, Vellore, Tamil Nadu 632014 India

**Keywords:** Electric vehicle, High voltage battery, Indian drive cycles, Battery energy assessment, Driving range, Electrical and electronic engineering, Batteries

## Abstract

The constant advancements in the research and development society of vehicle manufacturing made the customer’s attention towards EV ownership due to the better economic profile in the view of maintenance and operations. Even though the vehicles have better features the performance of the EV can be estimated with the consideration of the designed drive cycle for the region. According to that the procurement of EVs should be in the approved range of drive cycles with the concern of application. Based on that the article has concentrated on the performance of EV in the view of driving range and energy consumption of the battery for passenger transport applications. To observe the operational attributes of EV the assessment methodology was implemented for three Indian drive cycles (IDC) specifically on IUDC, MIDC, and IHDC. The quantitative assessment has been applied for the specifications of a 106-kW drive powered by the 30.2 kWh battery EV in the operational sequence model of three driving scenarios (aggressive, moderate, and slow) for a distance of 100 km with a four-wheel structure. To provide the vehicle statistics the assessment attempts the execution of performance parameters pictorially with the logistics of software evaluations for a particular average speed range of 21.2–50.02 km/h. From the assessment, it can be realized that the energy consumption of batteries is allowable in the range of 0.129–0.171 kWh/km for EV four-wheelers.

## Introduction

Vehicle emissions of pollutants are a major concern in metropolitan areas, as they harm air quality and people’s health^1^. The automotive sector is swiftly shifting from vehicles with combustion engines to battery-powered vehicles, encompassing hydrogen-powered fuel cells, electric vehicles (EVs), and hybrids. However, rising sales of electric vehicles are driving up demand for batteries, continuing the upward trend that has been seen in recent years. Compared to 2022, The requirement for Batteries for electric vehicles rose by 40% to more than 750 GWh by 2023. On the other hand, the annual growth rate was marginally lower than it was in 2021–2022. 95% of this increase can be ascribed to EVs. 95% of the rise in demand for batteries connected with EVs globally was attributable to increased EV sales, and the final 5% originated from larger typical battery dimensions due to an increasing approximate fraction of SUV sales among EVs^2–4^. Since Li-ion batteries have a remarkable energy and power density that leads to improved range and acceleration capabilities, they are in high demand as an energy source for electric and hybrid vehicles^5^. Various factors such as temperature, charging strategies, vehicle utilization trends, and mass of the vehicle have an impact on the lifespan and efficacy of electric vehicle batteries. The thermal variable takes into account both the external temperature and the heat generated internally within the energy pack and is contingent upon the battery’s consumption behavior^6^. Battery packs are often designed to operate under a wide range of temperatures. Any temperature beyond the manufacturer’s specified ideal working range will reduce the battery’s lifespan. Elevated temperatures can expedite chemical processes inside the battery, resulting in accelerated breakdown of internal components and diminished longevity. For each 10 °C increase in temperature, the battery’s useful life may be reduced by half due to expedited aging. Extended exposure to great heat can elevate the danger of thermal runaway, presenting significant safety issues. Conversely, low temperatures can diminish battery capacity and elevate internal resistance, complicating the battery’s charging and discharging efficiency. Temperature-induced fluctuations can lead to substantial deviations from the anticipated performance and safety of the battery. Similarly, the process of deep draining and rapid charging can expedite the degradation of batteries, ultimately reducing their lifespan. The utilization of batteries is a key factor that significantly impacts their longevity and efficiency. The degradation of battery cell capacity is commonly studied through the use of electrochemical equations, mathematical models of cell impedance, or empirical methods in battery performance and degradation evaluations. Nevertheless, these studies fail to take into account the practical use of batteries during real-world driving scenarios. Driving cycles provide an estimate of fuel efficiency, pollutants, and driving distance, which makes them useful for comparisons. Nevertheless, real-world driving conditions and their effects on emissions and fuel consumption are not well correlated, particularly when driving patterns are taken into account. Driving conditions in the actual world are very different from test conditions, which leads to noticeable differences in emissions, fuel efficiency, and driving distance^7^. The list of long-range EVs in India 2024 is shown in Table 1^8^. One frequent method to assess various user driving patterns and battery performance is to compare the battery performance data with typical drive cycles. The literature describes the use of a range of typical drive cycles to evaluate the performance of EVs and their subcomponents. However, there is currently little research on four-wheeler EV drive trains in the literature. To reflect the typical driving habits of the relevant areas, specific to a nation typical drive cycles are available. The Internationally Standardised Light Vehicle Examination.

**Table 1:** List of long-range EVs in India 2024^11^.

Name of the EV	Driving range (km)	Battery pack energy (kWh)	Charging time (mins)	Motor power (PS)
Tata Nexon EV Max	453	40.5	56	143
Mahindra XUV 400 EL	456	39.4	50	150
MG ZS EV	461	50.3	60	176
Tata Tiago EV	315	26	60	75
Mercedes Benz EQS	857	107.8	30	523
Kia EV6	708	77.4	18	229
BMW i4	590	83.9	60	340
Hyundai Kona EV	452	39.2	57	136
BMW i7	625	101.7	34	544
BYD Atto 3	521	60.48	50	204
BYD E6	521	60.48	50	204
Hyundai Ioniq 5	631	72.6	18	217

Cycle (WLTC) is a worldwide utilised drive cycle for testing light automobiles. ARTEMIS (Assessment and Reliability of Transport Emission Models and Inventory Systems) is the standard driving cycle used by European countries comprising different drive scenarios covering rural, urban, and freeway roads. UDDS (Urban Dynamometer Driving Schedule) represents the city driving conditions in the United States.

These are distinguished by varying top and bottom speeds, acceleration, and stopping periods that represent traffic patterns and road conditions. The automotive industry has frequently employed drive cycle-based techniques to assess fuel usage and conduct emission testing^1^.

In^2^, researchers examined how the drive cycle affects the component sizing of an electric car with a parallel configuration. EV battery performance might vary significantly based on driving conditions. A technique based on drive cycles can be used to assess these performance metrics in relation to the driving circumstances. Analyses and comparisons based on drive cycles that are comparable to the work suggested have been published in^3^.

The study described in mostly employs size reduction to analyse hybrid electric vehicle performance metrics based on several driving cycles techniques. Multi-criterion optimisation techniques are used to rank driving cycles based on how fuel-efficient they are. The analysis’s findings are verified by means of an intelligent power split control method based on genetic algorithms.

The battery’s efficiency assessment for the chosen urban drive cycles that match to a two-wheeler electric vehicle (EV) battery was described in the paper in^3^. The proposed work is unusual in that it takes into account the vehicle specifications that correlate to an LEV in the Indian market and analyses the battery endurance for both urban and highway drive cycles on Indian roads. For the purpose of comprehending battery usage behaviours and creating efficient energy-saving systems for an EV, battery efficiency parameters like temperature, SOC (state of charge deterioration), and consumption of energy are highly relevant. These studies should include models of the vehicle dynamics, battery packs, motors, power train control sets, and models of how the driving behavior of the user influences battery performance^1^.

This research primarily contributes to a system-level design and model of a four-wheeler electric vehicle drive train built with motors, a Li-ion battery pack, vehicle dynamics, and power train controllers. This drive train model has been used to assess battery performance under various driving conditions and user behaviors. By changing the speed trace of the actual drive cycles, the velocity scaling technique models additional driving characteristics of the user^9^. The variations of the energy-saving pack in the EVs are dependent on variable factors of SOC, temperature development, and energy consumption, which can be useful to understand the driving pattern of the owner at different altitudes^10^.

As a result of this analysis, the ideal speed range under each selected driving cycle to reduce battery energy consumption has been determined. Determining the ideal speed range and measuring battery performance parameters using simulated drive cycle behavior—with a focus on a four-wheeler electric vehicle drive train—are two of this work’s most novel discoveries^9^. By reducing energy use and battery deterioration, these discoveries may improve battery pack performance. While the general description of vehicle movement is provided in “Motion and dynamic equations for vehicles” section of Motion and Dynamic Equations for Vehicles, “Methodology used in EV drive train system” section covers the methodology and EV drive train system, including all of its parts, and “Overview of the PMSM-based EV powertrain system’s control technique” section discusses the Assessment of EV battery performance for diverse IDC inside the electric vehicle drive train. “Performance analysis of the Li-ion battery for EV drive pattern of various drive cycles of Indian roads” section covers the results and discussions, and the section that concludes comes next.

### Literature review

This section presents a classification of driving cycles along with a concise review of current methodologies for developing representative driving cycles. The research demonstrates that the provided methodology enhances prior driving cycle advancement approaches.

In 1985, using data obtained from Mumbai, Chennai, Bengaluru, and Pune, the Automotive Research Association of India established an official Indian Drive Cycle (IDC). The greatest speeds of acceleration and deceleration for the 108-s cycle were 0.65 and 0.63 m/s^2^, respectively, while the mean speed was 21.9 kmph. The modified IDC (MIDC), which has a highest acceleration of 0.833 m/s^2^, a peak deceleration of 1.389 m/s^2^, and a mean speed of 59.3 kmph, was approved in 2000 (Ministry of Road Transport & Highways, 2000)^12^

The performance parameters that exist in all vehicle speeds (such as travelling, stationary, and accelerating) across every zone are thought to be represented through these drive cycles. Nonetheless, the driving qualities varied greatly between cars and geographical areas.

As a result, the IDC or MIDC might not accurately reflect the driving attributes of all kinds of cars and trucks, particularly goods vehicles, which are usually seen on roadways with lower speeds, greater weights, and slower rapid acceleration and deceleration. Additionally, these shortcomings of normal drive cycles result in inaccurate network-level pollution estimations. To ascertain the present consumption of energy and emissions levels, it is imperative that real-world drive cycles tailored to goods vehicles be developed.

The following vehicle operating situations make up a normal drive cycle: cruising, creep, acceleration, braking, and idle. Drive cycles typically last between 10 and 30 min, which is both feasible and economical for data gathering while being adequate to capture the variances of real-world driving parameters.

Automobile design and law typically use conventional worldwide driving cycles, referred to as ‘legislative’ cycles. Though, practical driving habits and, consequently, driving cycles varies significantly across towns and areas due to variations in network condition, road conditions, municipal and regional characteristics, socioeconomic and historical environment, and type of vehicle. Because of this, conventional global cycles fail to sufficiently reflect context-dependent driving behaviours and transients, nor do they depict driving situations found in the actual world^13^.

Apart from being categorised as legislative or non-legislative, driving phases are also categorised as “transient” or “modal” based on their development. While modal cycles are created from a series of steady velocity and acceleration states, or “modes,” transient cycles are created using on-road vehicle data.

The literature generally divides approaches for creating transient driving cycles into five major types.

Numerous of them entail "micro-trips," which are commonly described in the literature^14^ as parts of a journey in which the car stops and starts again. When assessing a vehicle’s pollution performance straight out of the drive cycle, multiple investigations have classified micro-trips as trip portions when the engine RPM drops from zero to zero. Nevertheless, vehicle speed is employed in this investigation because engine RPM data was not available.

Random selection-based (RS), segment-based (SB), monte Carlo Markov chain (MCMC)-based, clustering analysis-based (CA), and optimization-based (OB) approaches are the five categories^15^.

Using a variety of methods, we can create a drive cycle for any route, highway, city, area, or country. The following steps are necessary for a drive cycle to grow: Path selection, data collecting, data organisation, and drive cycle construction are the first three steps^13^.

### Key features of the IDC

The driving cycle (DC) serves as a fundamental instrument for automotive certification; nevertheless, standard DCs fail to represent the actual driving circumstances of certain regions, resulting in inaccuracies in evaluations of performance. The Indian Drive Cycle (IDC) approach is a methodology used to evaluate the performance of electric vehicles (EVs) under typical Indian driving conditions. This approach involves the use of specific driving cycles that reflect the unique traffic, road, and climatic conditions in India. Here’s a detailed explanation of the IDC.

- Standardization for certification: The IDC was established as a pioneering standard for vehicle certification in India, focusing on safety and emissions. This standardization is crucial for ensuring that vehicles meet regulatory requirements while reflecting the unique driving conditions of the region.
- Driving conditions representation: The IDC aims to capture the varied driving scenarios encountered on Indian roads, which include a mix of urban congestion, rural driving, and highway conditions. This is essential for accurately assessing vehicle performance and emissions, as traditional driving cycles may not adequately represent these diverse conditions.
- Energy consumption assessment: The IDC approach allows for the evaluation of energy consumption and battery performance in EVs. By simulating real-world driving patterns, the IDC helps in understanding how different driving behaviors impact the efficiency and longevity of electric vehicle batteries.
- Adaptation to local needs: The IDC is tailored to the specific needs and challenges of the Indian market, making it relevant for local manufacturers and policymakers.

This localized approach is vital for promoting the adoption of EVs in India, as it provides insights into how vehicles can be optimized for the unique driving conditions present in the country**.**

## Methodology used in EV drive train system

The assessment of battery efficiency amidst various user driving behaviors necessitates a blueprint for the electric vehicle’s four-wheeled powertrain. The essential elements of the drive system consist of a suitable battery pack for four-wheeled EVs, along with the motor, controller, and the intricacies of vehicle dynamics.

An example of the Battery Electric Vehicle (BEV) power system architecture opens the methodology. The main components of this architecture are a power battery, a DC/DC converter, and a motor. To power the electric vehicles, the DC/DC converter amplifies the electric current from lithium batteries and directs it into a three-phase AC motor.

Driving Cycle Assessment: Using three distinct Indian Drive Cycles (IUDC, MIDC, and IHDC), the study aims to assess how well electric cars function. These cycles are intended to replicate actual driving circumstances, which are crucial for comprehending how different elements impact battery life and energy usage.

**Fig. 1:**
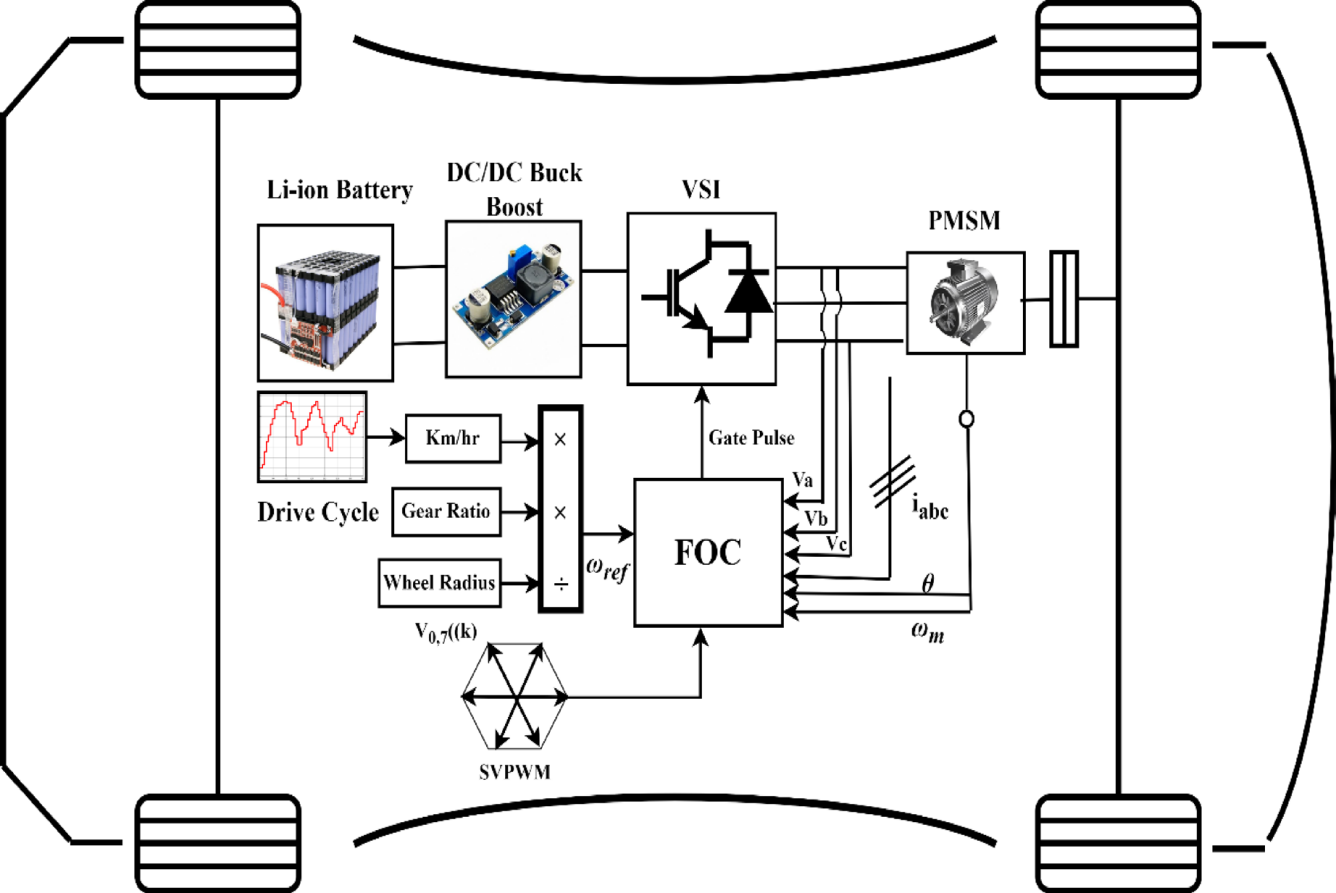
Block diagram of EV Powertrain system.

**Fig. 2:**
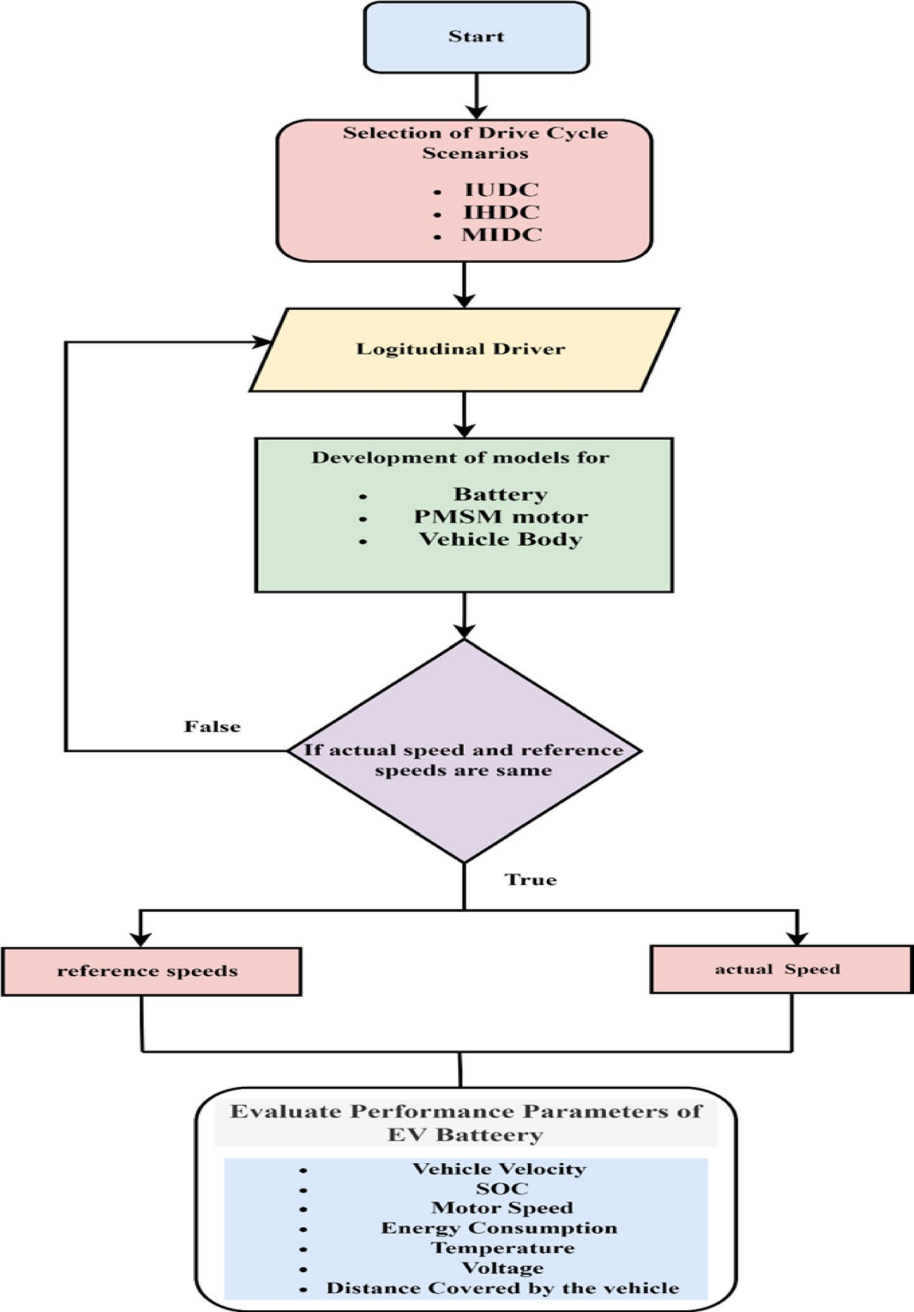
flow chart for electric vehicle model driving behaviour with IDC scenarios.

As illustrated in Fig. 1, the architecture of the power system for BEVs predominantly consists of a motor, a DC/DC converter, and a power battery^16^. Following amplification at DC/DC, the electric current originating from lithium batteries is directed into a three-phase AC motor, which powers electric vehicles (EVs)^17^. The electric vehicle powertrain, crafted through simulation and utilizing the driving cycle as its guiding velocity, is depicted in Fig. 2. The driving cycle, a collection of data points mapping the vehicle’s speed over time has been considered as a primary input and provided to the model. This driving cycle is dispatched to the longitudinal driver block, which orchestrates commands for both braking and acceleration by harnessing velocity feedback alongside the driving cycle in a predictive control manner.

Every component of the model’s intricate systems draws its energy from a datasheet battery^16^. This power source, among its myriad functions, provides the voltage and current of the battery while simulating a lithium-ion battery model that reflects discharge traits across various temperatures. The Motor Drive module, which is elegantly divided into two distinct subsystems—Motor Control and Regenerative Braking—gathers the battery voltage, vehicle speed feedback, and commands for both acceleration and deceleration. To initiate the torque calculation, the vehicle speed must first be transformed into motor RPM. Regenerative Braking takes in the motor speed, vehicle speed, state of charge (SoC), and braking directive^17^. It subsequently sends forth the conclusive brake instruction alongside the revitalized torque. The motor controller captures the renewed torque, rotational speed, and battery charge level^18^. The final manifestation of the motor torque is then conveyed to the transmission system, wheels, and braking mechanism to generate the vehicle’s velocity.

The driver segment of the model receives this output velocity and employs forecasting techniques to correct any discrepancies in speed. The existing motor data is harnessed to craft a mesmerizing contour plot that illustrates the motor’s efficiency. The specifications for the electric vehicle alongside the details of the high-voltage battery pack are encapsulated in Tables 2, 3, and 5^19^. The following are the comprehensive methodological procedures used in EV drive train model.

1. Simulation of real-world situations: To evaluate the operating characteristics of electric vehicles, the technique highlights the significance of modeling real-world driving situations. This method is essential because it makes it possible to assess battery performance more precisely than with conventional test conditions, which could not accurately represent real-world driving situations. Figure 2 shows flow chart for electric vehicle model driving behaviour with IDC scenarios.
2. Quantitative evaluation: A 30.2 kWh battery-powered electric car model is the subject of a quantitative evaluation. The study looks at how much energy is used when driving 100 kms in three different driving scenarios: aggressive, moderate, and leisurely. This quantitative method offers a thorough comprehension of how driving habits affect battery performance.
3. Parameters of performance visualisation: Using software evaluations to execute performance parameters is part of the technique. This enables vehicle statistics to be represented graphically, which facilitates data analysis and the derivation of conclusions regarding energy efficiency and consumption at different speeds (21.2–50.02 km/h).
4. Real-world application: The methodology seeks to offer insights that help direct manufacturers in creating more efficient electric vehicles by concentrating on real-world driving situations and utilizing quantitative assessments. Given India’s distinct driving conditions and consumer demands, this is especially pertinent.

**Table 2:** PMSM specifications:

Parameter	Ratings	Units
Type of motor	PMSM	
Electric motor power	106	(kW)
Electric motor torque	245	(Nm)
Stator Resistance (${R}_{s}$)	6.5	m $\Omega$
Stator inductance (${L}_{q}$, ${L}_{q}$)	8.35	mH
Motor inertia	0.089	Kg m^2^
Number of poles	4	
PM magnet flux	0.176	Wb

**Table 3:** Electric vehicle specifications.

Parameter	Ratings	Units
Length * width * Height	3993*1811*1606	(mm)
Wheelbase	2498	(mm)
Kerb weight	1400	(kg)
Number of passengers	5	
Passengers weight	70	(kg)
Payload weight	50	(kg)
Total mass of the vehicle	1800	(kg)
Ground clearance	205	(mm)
Boot space	350	(lt)
Wheel radius	0.34	(m)
Wheel/tires	R 16 215/60 LRR	
Grade angle	0.0872	radians
Drag coefficient	0.24	
Gear ratio	9.1	
Frontal area	2.91	(m^2^)
Acceleration due to gravity	9.81	m/s^2^
Rolling resistance coefficient	0.015	
Range	100	(Km)

**Table 4:** Switching states of SVPWM.

(S_1_, S_2_, S_3_)	Voltage vectors	(S_1_, S_2_, S_3_)	Voltage vectors
(0,0,0)	${V}_{0}$= 0	(0,1,1)	V_4_ = $-\frac{2}{3}{V}_{dc}$
(1,0,0)	${V}_{1}$= 0	(0,0,1)	${V}_{5}=-\frac{1}{3}{V}_{dc}-j\frac{\sqrt{3}}{3}{V}_{dc}$
(1,1,0)	${V}_{2}=\frac{1}{3}{V}_{dc}+j\frac{\sqrt{3}}{3}{V}_{dc}$	(1,0,1)	${V}_{6}=\frac{1}{3}{V}_{dc}-j\frac{\sqrt{3}}{3}{V}_{dc}$
(0,1,0)	${V}_{3}=\frac{1}{3}{V}_{dc}+j\frac{\sqrt{3}}{3}{V}_{dc}$	(1,1,1)	${V}_{7}=7$

## Motion and dynamic equations for vehicles

The principles underlying vehicle design are fundamentally rooted in the core concepts of physics, specifically the second law of motion articulated by Sir Isaac Newton. As per Newton’s second principle, the total force influencing an object defines its rate of acceleration in a directly corresponding fashion. An object will therefore experience acceleration when the cumulative force applied to it is non-zero. A variety of forces are exerted upon a vehicle; the motion of the vehicle is governed by Newton’s second law through the net or resultant force. The force necessary to propel the vehicle forward is generated by its propulsion system. This force produced by the propulsion system empowers the vehicle to surmount resistive forces, which arise from gravitational pull, aerodynamic resistance, and tire friction. The speed of the motor vehicle is contingent upon^20^. The output power of the propulsion unit, the condition of the roadway, the aerodynamic characteristics of the vehicle, and its mass in accordance with Newton’s second law of motion are all elucidated.

A comprehensive overview of the vehicle’s motion is presented in the below section which means the resistance encountered by vehicles, and the dynamic equations including the adhesion of tires to the surface and the maximum tractive force.

### Design elements for the electric vehicle system

**Fig. 3:**
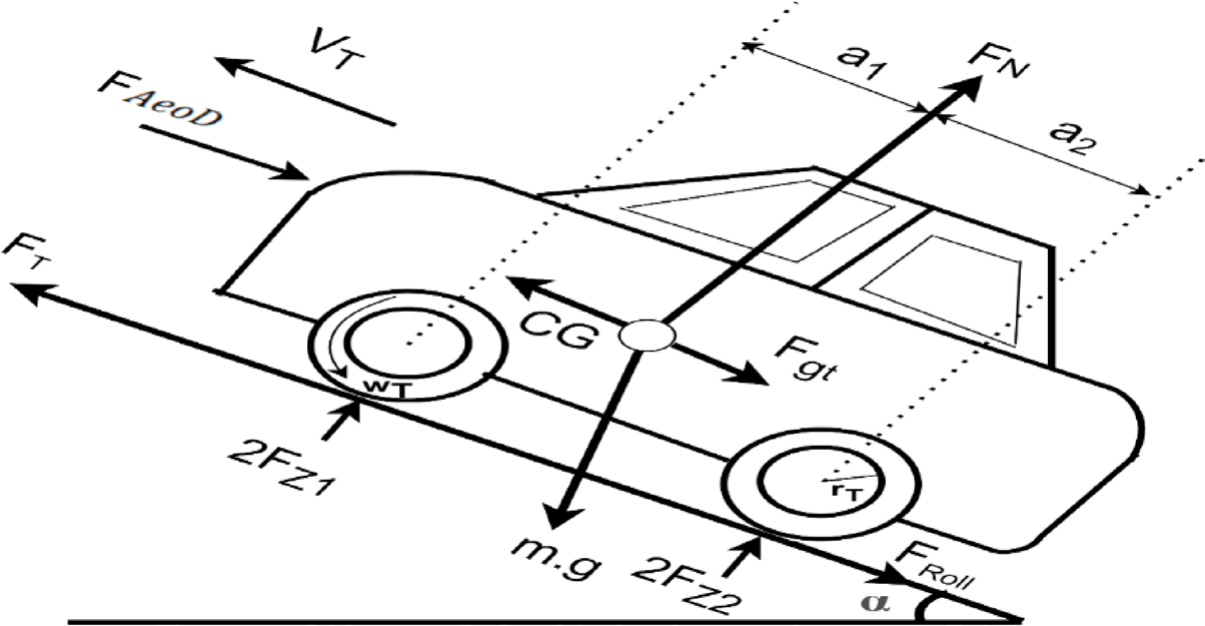
Forces applied to an electric vehicle in motion. Where, FT = Tractive force (propulsion force) acting at the driven wheels. VT = velocity of the vehicle in alignment with the direction of the road, Fgt = cumulative effect of the gravitational force, FRoll = rolling resistance of the vehicle tires, FAeoD = aerodynamic drag^18^. CG: Center of Gravity of the vehicle. m g: Total gravitational force acting downwards (where m is mass and g is acceleration due to gravity). 2FZ1: Normal force acting on the front wheels. 2FZ2: Normal force acting on the rear wheels. WT: Wheel torque. α: Angle of inclination of the road. a1: Horizontal distance from the center of gravity (CG) to the center of the front axle. a2: Horizontal distance from the center of gravity (CG) to the center of the rear axle. rT: Radius of the rear wheel.

Investigating the influences on a car’s movement provides a comprehensive understanding of its operational dynamics. The forces applied to a vehicle ascending an incline are illustrated in Fig. 3. The tractive force (${\text{F}}_{\text{T}}$) Present at the interface between the tire contact surface and the road surface propels the vehicle in a forward direction. This tractive force (${\text{F}}_{\text{T}}$) is generated by the powertrain and transmitted to the driving wheels through the transmission system and the final drive assembly.

As the object progresses, it encounters a resistive force that endeavours to obstruct its speed^21^.

- Rolling resistance
- Aerodynamic drag
- Uphill resistance

1 $$F_{T} = F_{gt} + F_{Roll} + F_{AeoD}$$

2 $$F_{gt} = m.g.\sin \theta$$

3 $$F_{Roll} = mg\cos \theta \left( {R_{c0} + R_{c1} v_{t}^{2} } \right)$$

4 $$F_{AeoD} = \frac{1}{2}\rho D_{c} A_{f} \left( {v_{t} - v_{0} } \right)^{2}$$

Where, ${v}_{0}$ is defined as positive for a headwind and negative for a tailwind.

This modification accurately reflects the influence of wind direction on the relative velocity between the vehicle (${v}_{t}$) and the air. A headwind (positive ${v}_{0}$) increases the relative velocity $\left({v}_{t}-{v}_{0}\right)$, resulting in higher drag and a subsequent reduction in traction. Conversely, a tailwind (negative ${v}_{0}$) decreases the relative velocity, lowering drag and increasing the available traction force.

The coefficients of rolling resistance are denoted as ${\text{R}}_{\text{c}0}$ and ${\text{R}}_{\text{c}1}$. The gradient in degrees is represented by $\uptheta$. The acceleration due to gravity is denoted as g. The air density is represented by ρ. ${\text{D}}_{\text{c}}$ represents the coefficient of drag. ${\text{A}}_{\text{f}}$ represents the frontal area. Lastly**,** ${\text{v}}_{0}$ represents the recorded headwind velocity ahead of the vehicle. Equation (2) articulates the correlation between the aerodynamic force and the previously defined constants and variables. Figure 3 is widely referred to as a free-body diagram or point mass diagram. It depicts the forces acting upon the electric vehicle while it is traversing a roadway.

The comprehensive effective mass of the vehicle is ${\text{M}}_{\text{eff}}$ ^22^ constituted by the aggregate of the vehicle’s mass and the equivalent mass attributable to the inertia of both the motor and the wheels.

5 $${\text{M}}_{{{\text{eff}}}} = {\text{M}} + \frac{{4.{\text{J}}_{{\text{t}}} }}{{{\text{R}}_{{\text{C}}}^{2} }} + \frac{{{\text{J}}_{{{\text{mi}}}} }}{{{\text{r}}_{{\text{d}}}^{2} {\text{R}}_{{\text{C}}}^{2} }}$$

6 $${\text{I}}_{{\text{C}}} = { }\frac{{({\text{F}}_{{{\text{gt}}}} + {\text{F}}_{{{\text{Roll}}}} + {\text{F}}_{{{\text{AeoD}}}} ){\text{V}} \times \eta_{{\text{Q}}} }}{{{\text{V}}_{{\text{n}}} \times \eta_{{\text{D}}} \times { }\eta_{{{\text{motor}}}} \times \eta_{inverter} }}.$$

7 $${\text{I}}_{{\text{D}}} = { }\frac{{({\text{f}}_{{{\text{gt}}}} + {\text{f}}_{{{\text{Roll}}}} + {\text{f}}_{{{\text{AeoD}}}} ){\text{V}}}}{{{\text{V}}_{{\text{n}}} \times {\upeta }_{{\text{D }}} \times {\upeta }_{{{\text{motor}}}} \times {\upeta }_{inverter} }}$$

where, ${\upeta }_{\text{motor}}$ is the efficiency of the electric motor, ${\upeta }_{inverter}$ is the efficiency of the inverter, M = vehicle mass (kg), ${\text{J}}_{\text{t}}$ = tire inertia (kg m^2^), ${\text{J}}_{\text{mi}}=$ motor inertia in terms of mass (kg m^2^), ${\text{R}}_{\text{c}}$ = tire effective rolling resistance coefficient, ${\text{r}}_{\text{d}}$ = gear reduction ratio.

Where, ${\text{I}}_{\text{C}}$ = Battery current at the condition of charging (A)^23^, ${\text{V}}_{\text{n}}$ = Battery nominal voltage (V), V = Vehicle velocity (km/hr) ${\upeta }_{\text{Q}}$= Performance of battery charge. ${\text{I}}_{\text{D}}$= Battery current at the condition of discharging (A), and ${\upeta }_{\text{D}}$= Performance of battery discharge. The vehicle dimensions and forces are the deciding factors in the sizing of the battery and motor. The developed torque of the wheel has been evaluated by Eq. (9) according to the force exerted data from Eq. (2).

8 $${\text{T}}_{{\text{t}}} = {\text{F}}_{{\text{T}}} {\text{t}}_{{\text{R}}}$$

where ${T}_{t}$ = tire torque (Nm), ${t}_{R}$ = tire radius (m)

9 $${\text{t}}_{{\text{w}}} = { }\frac{{{\text{V}} \times 60}}{{2\pi \times {\text{t}}_{{\text{R}}} }}$$

where ${t}_{w}$= tire speed (rad/s),

10 $${\text{G}}.\;{\text{R}} = \frac{{T_{t} }}{{T_{m} }}$$

where G.R = Gear ratio, ${\text{T}}_{\text{t}}$= tire torque (Nm), T_m_ = Motor torque (Nm).

Torque and speed of the motor with the concern of gear ratio and transmission efficiency

11 $${\text{T}}_{{\text{m}}} = \frac{{{\text{T}}_{{\text{t}}} }}{{{\text{G}}.{\text{R}} \times { }\eta_{{\text{T}}} }}$$

where ${\eta }_{T}$ = Transmission efficiency.

motor output power in (W)

12 $$P_{out, m} = T_{m} \times W_{m}$$

where ${P}_{out, m}$ = Motor output power (W).

13 $${\text{B}}_{{\text{P}}} = \frac{{{\text{P}}_{{{\text{eff}}}} }}{{{\text{inv}}_{{{\text{eff}}}} }}$$

where ${\text{B}}_{\text{P}}$ = Battery power (kWh), ${\text{inv}}_{\text{eff}}$ = Inverter efficiency

14 $${\text{B}}_{{{\text{Ah}}}} = \frac{{B_{P} }}{{V_{Dc} }}$$

where ${\text{B}}_{\text{Ah}}$ = battery capacity (Ah) and ${\text{V}}_{\text{Dc}}$ = input voltage of the battery (V)

The Range of Ev can be calculated

15 $${\text{Estimated}}\;{\text{range}}\;\left( {{\text{km}}} \right) = \frac{{{\text{B}}_{{{\text{Ah}}}} }}{{{\text{B}}_{{\text{P}}} }}$$

The PMSM drive is modelled in terms of a synchronously rotating d-q reference frame. The currents through the stator of the PMSM drive are expressed as follows:

16 $$\frac{{di_{d} }}{dt} = - \frac{{R_{s} }}{{L_{d} }} i_{d} + \frac{{L_{q} }}{{L_{d} }}w_{e} i_{q} + \frac{1}{{L_{d} }}u_{d}$$

17 $$\frac{{di_{q} }}{dt} = - \frac{{R_{s} }}{{L_{q} }} i_{q} - \frac{{L_{d} }}{{L_{q} }}w_{e} i_{d} - \frac{1}{{L_{q} }}\psi_{m} + \frac{1}{{L_{q} }}u_{q}$$

where ${i}_{d}$, ${i}_{q}$ = motor stator current, ${R}_{s}$ = motor stator resistance, ${L}_{q}$, ${L}_{q}$ = motor stator inductance in d-q reference frame, ${w}_{e}$ = electrical rotor speed, ${\psi }_{m}$ = flux linkage, ${u}_{q}$, ${u}_{d}$ = motor stator voltages.

### Brief description of 3-phase voltage source inverter

This paper considers feeding a PMSM with a two-level voltage source inverter (2L-VSI). Equations indicate that the resultant phase voltages of VSI are defined by the signals that switch and the voltage across the DC bus (V_dc_).

$$\begin{aligned} & {\text{V}}_{{\text{a}}} = {\text{S}}_{{1}} {\text{V}}_{{{\text{dc}}}} \\ & {\text{V}}_{{\text{b}}} = {\text{S}}_{{2}} {\text{V}}_{{{\text{dc}}}} \\ & {\text{V}}_{{\text{c}}} = {\text{S}}_{{3}} {\text{V}}_{{{\text{dc}}}} \\ \end{aligned}$$

**Fig. 4:**
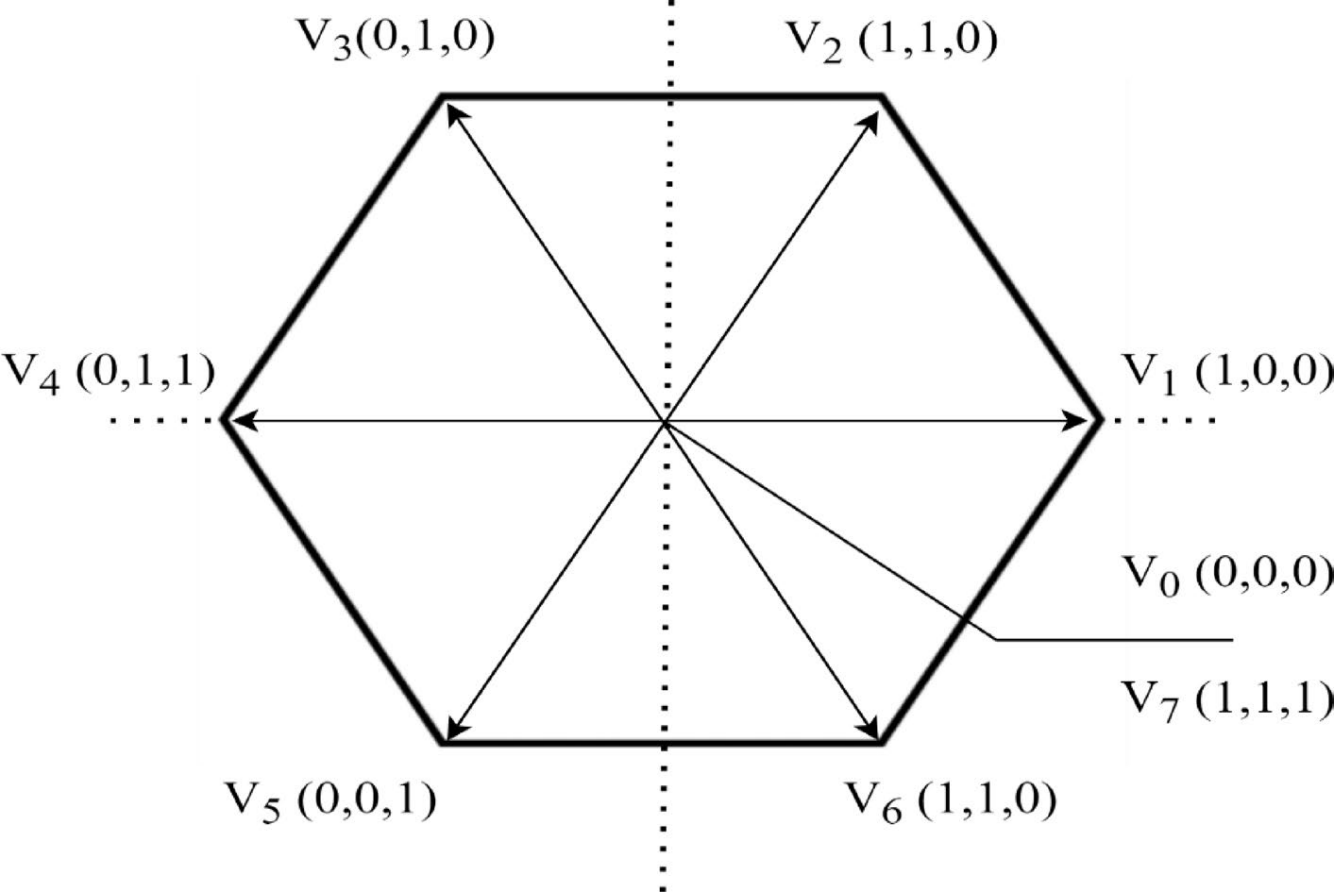
Voltage vector using the standard VSI SVPWM.

Switching levels and their respective voltage vectors are illustrated in Table 4 and Fig. 4. It includes eight potential switching states, comprising two zero voltage vectors (V0, V7) and six non-zero voltage vectors (V1, V2, V3, V4, V5, V6).

**Table 5:** Details about high-voltage battery pack.

Parameter	Ratings	Units
Battery pack energy	30.2	(kWh)
Battery pack voltage	320	(V)
Battery capacity	94.375	(Ah)
Nominal voltage	3.2	(V)
Pack weight	260	(kg)
Cylindrical cell (IFR32135-15Ah)	32 mm diameter, 135 mm Height	
Number of series cells	100	
Number of parallel cells	6	
Total cells	600

## Overview of the PMSM-based EV powertrain system’s control technique

Over a large speed range, the FOC demonstrates exceptional precision and outstanding dynamic performance. To attain the highest torque possible, the stator and rotor fields of AC machines are not perpendicular to one another. The motive of the FOC method is to align the resultant magnetic field created by the stator current to be orthogonal to the permanent magnetic field of the rotor. Using Id = 0 as the FOC approach to control surface mount PMSM gives the regulation system several benefits, including low impulse and superior torque characteristics.

This approach, also known as the vector control method, facilitates the decoupling of torque and flux control of AC machines. FOC converts the three-phase stationary input current components Ia, I_b_, and Ic into the two-phase rotating reference frames I_d_ and Iq. Here, Iq and Id are the stator current components that produce torque and flux, respectively. Since this control method is rotor flux aligned, the Id component of stator current should be zero to generate the highest torque because the Id axis aligns with the rotor flux axis.

Flux weakening is employed in conjunction with Field-Oriented Control (FOC) to regulate the speed and torque of the Permanent Magnet Synchronous Motor (PMSM). The PMSM generates its rated torque at lower speeds using nominal stator current and magnetic flux in the constant torque band. When the motor reaches its base speed, the generated stator voltage and output power rise in proportion to its rotating speed. The base speed is established by the greatest voltage that the Voltage Source Inverter (VSI) can provide.

**Fig. 5:**
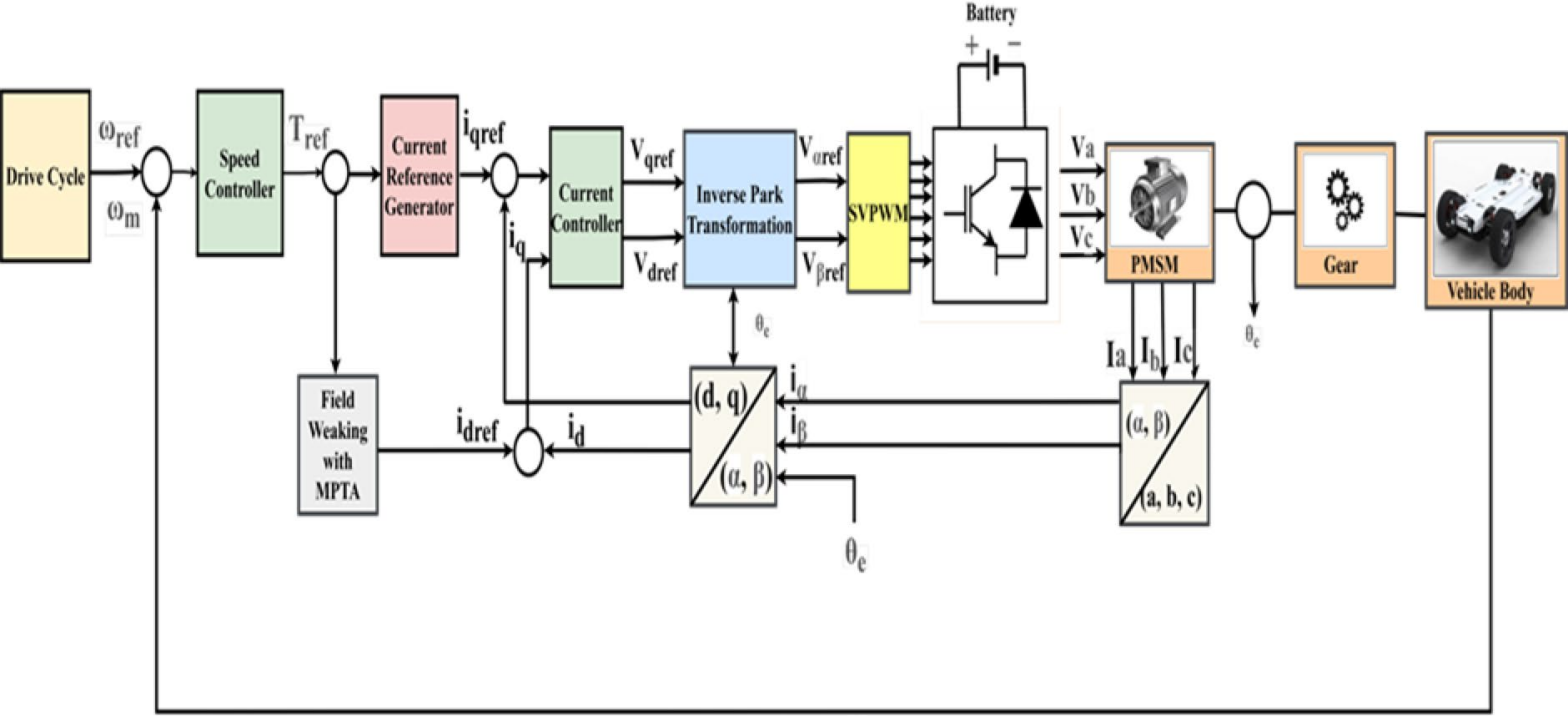
Block diagram for the assessment of various drive cycles in the vehicle-electric powertrain system.

Flux weakening is applied to cross this basic limit and enter the constant power area. This technique reduces the rotor flux magnitude while maintaining the stator voltage at its rated limit. According to the fundamental relationship between power, torque, and speed, the available torque falls inversely with speed when the motor’s speed rises above the base speed to maintain a comparatively constant output power. The flux-weakening zone, also known as the constant power region, allows a motor to run faster than its base speed without going over the voltage limits. The test drive setup used to analyse battery performance in electric vehicles during different drive cycles, where this control mechanism is crucial, is shown in Fig. 5.

Field-Oriented Control (FOC) makes it possible to independently and dynamically control the magnetic flux and torque of the motor. During the flux-weakening period, the stator current’s direct-axis current (Id) component is purposefully reduced (made negative). A magnetic field created by this negative Id current opposes and weakens the permanent magnet flux that the rotor produces. This decrease in net magnetic flux ensures stable operation within the VSI’s voltage limitations by limiting the back electromotive force (back-EMF) produced by the motor at high speeds. This control approach is crucial for applications requiring efficient speed control over a broad range, such as high-speed industrial motors and battery-powered electric cars. In the d-q current plane, the control system may also employ a Maximum Torque Per Ampere (MTPA) trajectory to maximise efficiency.

The PMSM is controlled by FOC with estimated flux, and the torque production is usually mostly caused by the quadrature-axis current (Iq). For maximum efficiency, the direct-axis current (Id) should ideally be set to zero in the constant torque area. Nevertheless, Id is purposefully made negative in the flux-weakening zone. The motor’s maximum speed under this control scheme is restricted by the back electromotive force (EMF), the maximum stator voltage that can be obtained from the inverter, and the prescribed stator current rating. The back EMF produced by the motor may be greater than the provided voltage if the drive is running faster than its base speed, which could cause instability. This is addressed by driving the Id variable negative, which essentially lowers the rotor flux linkage and permits the PMSM to function above its base speed. This technique is specifically known as the field-weakening control strategy.

The PMSM’s characteristics and the Voltage Source Inverter’s (VSI) ability to produce the required flux weakening are used to calculate the reference value for Id (idref). However, with the field-weakening approach, the motor’s rated current restrictions and load requirements limit both the possible torque output and the reference value of Iq (iqref).

**Fig. 6:**
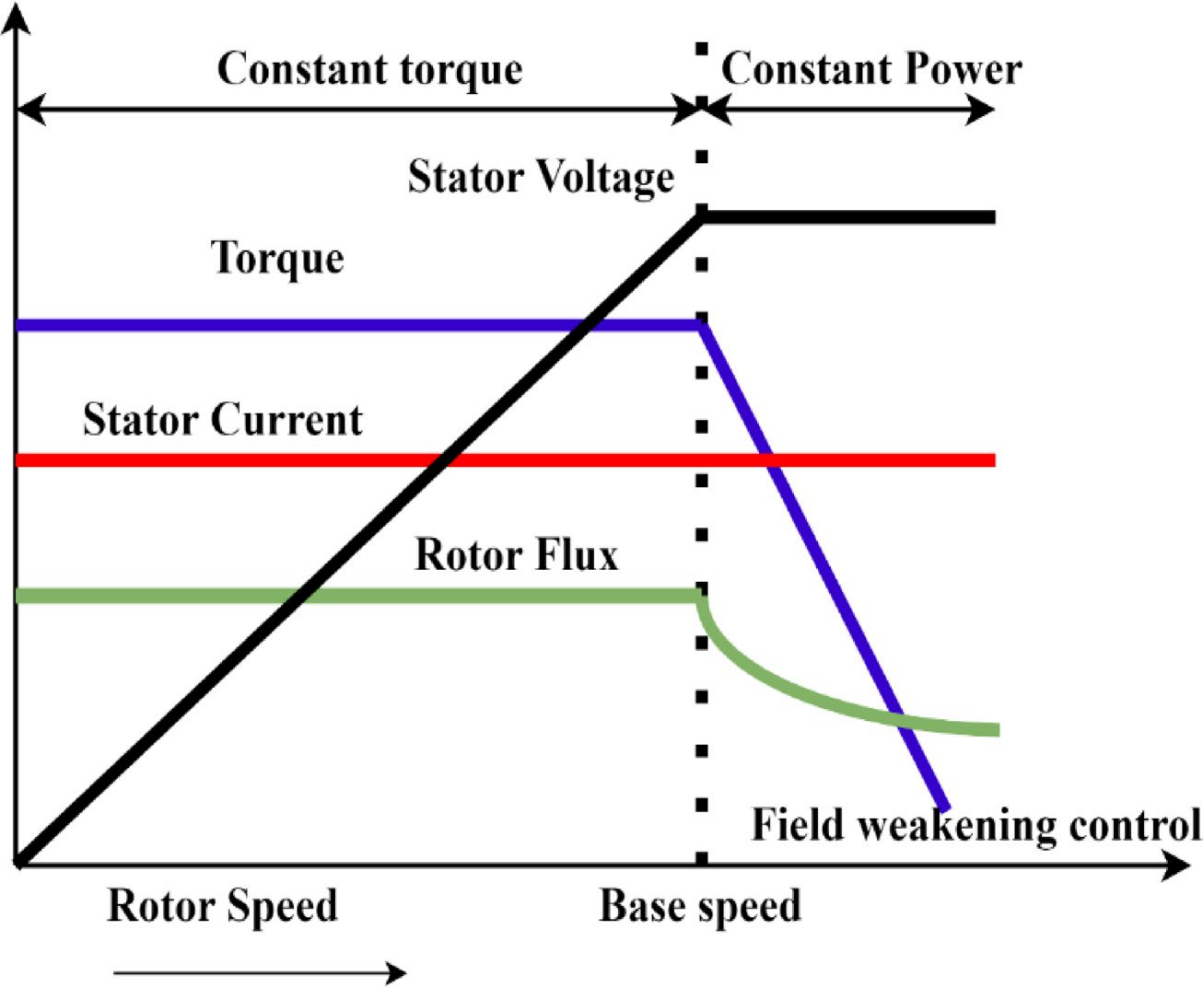
Optimal field weakening characteristics associated with PMSM.

As a result, as shown in Fig. 5, the motor runs in the constant torque domain until it reaches its base speed, after which it moves into the constant power sector, which is distinguished by a diminishing torque capacity. According to the FOC technique is used to manage the speed and torque of the PMSM are managed. The torque and speed characteristics of the suggested drive train system as a function of the motor’s field-weakening factor are graphically depicted in Fig. 6.

The PMSM’s characteristics and the Voltage Source Inverter’s (VSI) ability to produce the required flux weakening are used to calculate the reference value for Id (idref). However, with the field-weakening approach, the motor’s rated current restrictions and load requirements limit both the possible torque output and the reference value of Iq (iqref). As a result, as shown in Fig. 6, the motor runs in the constant torque domain until it reaches its base speed, after which it moves into the constant power sector, which is distinguished by a diminishing torque capacity. According to the FOC technique is used to manage the speed and torque of the PMSM are managed^24^. The torque and speed characteristics of the suggested drive train system as a function of the motor’s field-weakening factor are graphically depicted in Fig. 6.

Reducing or weakening the flux while maintaining a constant voltage at the specified value is necessary if the speed is to be increased beyond its maximum. Due to the torque’s inverse relationship with speed, the output power doesn’t change beyond the base speed^23^.

## Performance analysis of the Li-ion battery for EV drive pattern of various drive cycles of Indian roads

The Indian Driving Cycle (IDC) constituted the pioneering standard for vehicle certification in India, emphasizing safety and emissions. Nevertheless, the IDC fails to adequately represent the varied driving conditions encountered on Indian roads. The sequence consists of six driving scenarios, each extending for 108 s, maintaining an average velocity of 21.9 km/h across a span of 3.94 km. This speed does not accurately reflect conditions on rural roads or congested urban areas. In addition, the IDC overlooks the preconditioning idle time of diesel vehicles, which lasts for 40 s before the chassis dynamometer test and only considers minimal accelerations (< 0.65 m/s^2^) and engine loads. The cold start phase is additionally excluded^25^.

Power demand zones. To more accurately replicate driving conditions in urban environments, the Indian Urban Drive Cycle (IUDC) was created. The IUDC mimics the stop-and-go nature. The Modified Indian Driving Cycle (MIDC), sometimes known as the New European Driving Cycle (NEDC), was created to address these limitations. With a 90 km/h maximum speed limit, the ability to calculate vehicle emissions effectively is required. Real speed acceleration profiles in Indian cities are significantly higher than those required by the MIDC, with significant differences observed between the two. As a result, the MIDC underestimates emissions during these phases, especially NOx and other pollutants, since it does not appropriately represent high levels. The MIDC mimics idle conditions more accurately and offers wider speed profiles than other driving simulators. Over an 11-km span, the MIDC covers a variety of driving scenarios at a mean speed of about 33.6 km/h. Despite these developments, variations in traffic density, land-use patterns, road infrastructure, and traffic management pose challenges to the MIDCs of city traffic by making multiple stops, idling, and speed variations. This cycle incorporates the higher accelerations and decelerations common to driving in cities, providing a more realistic depiction of the driving conditions there. With a mean speed of almost 17 km/h, the IUDC travels 4.1 km overall. To replicate the circumstances of driving on highways, the Indian Highway Drive Cycle (IHDC) was also included.

**Fig. 7:**
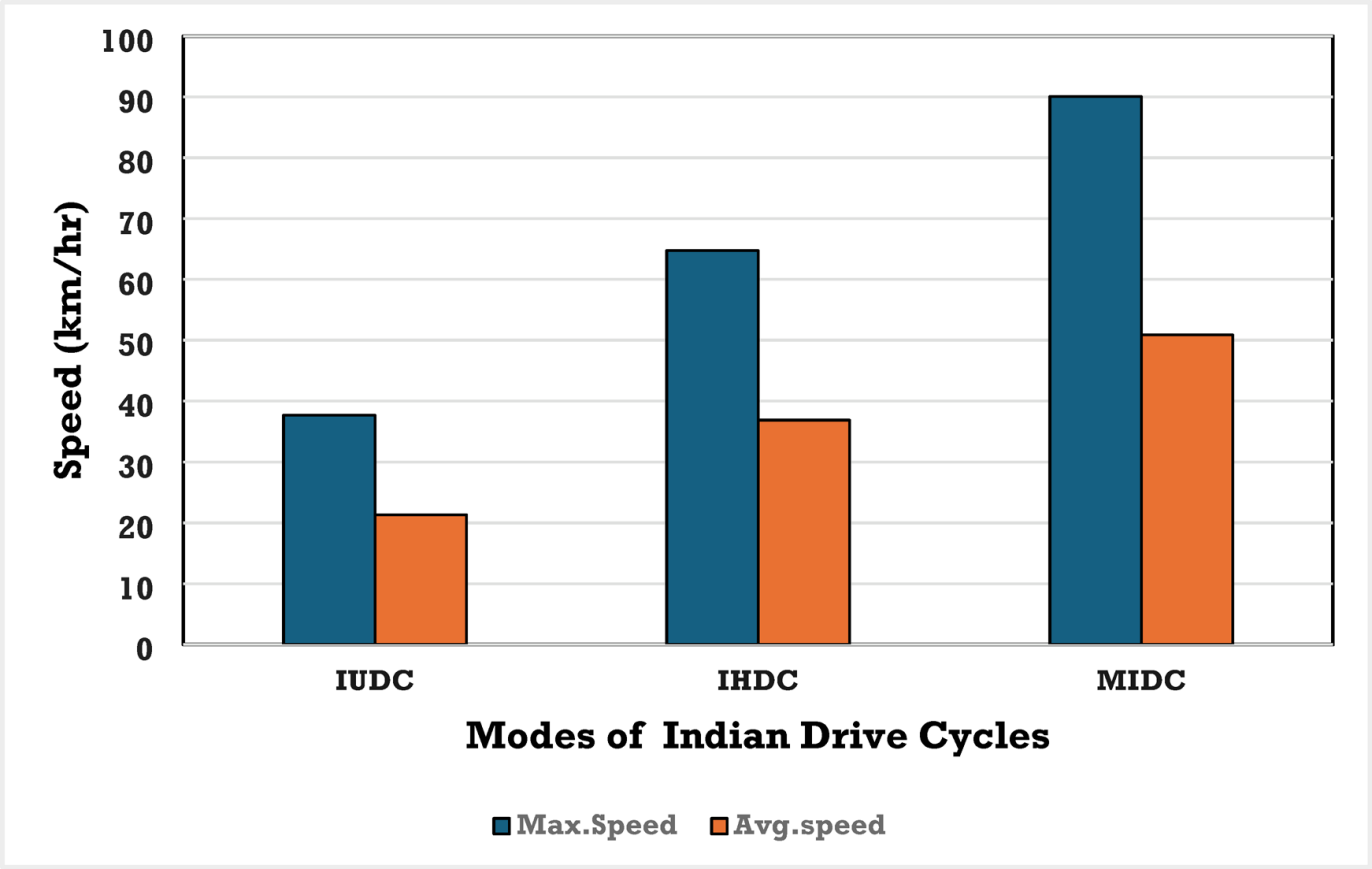
Max. speed/Avg. speed of the vehicle for Indian urban drive cycle/Indian highway drive cycle/modified Indian drive cycle^4^.

The IHDC mirrors the characteristics of highway traffic with its greater speeds and more consistent driving patterns. This cycle travels 10 km at a mean speed of about 60 km/h, with greater accelerations and decelerations to accommodate overtaking and varying highway traffic conditions. To offer a more precise assessment of vehicle emissions and ensure compliance with environmental regulations, future driving cycles must take these variations into account. The pattern of the drive cycles is a vital factor for the variations in the EV’s acceleration, deceleration, braking, stop time, and performance. The following parameters: kinetic intensity, average running time, average acceleration, average deceleration, average speed, average stop time duration, maximum speed, maximum acceleration, and percentage of time spent in acceleration, deceleration, and ideal mode metrics are the necessary considerations for the design of drive cycles. The Maximum and average speed of the EV for IUDC, IHDC, and MIDC are depicted in Fig. 7.

These factors are suitable for optimizing the powertrain components and quantifying the environmental impact of transportation, and vehicle size for better fuel consumption^26^. The EV operation is an intermittent rotational force generation in a motor for a variable state of momentum called a drive cycle. Speed and elevation are measured second by second during a normal drive cycle. The drive cycle is a vital input factor for the process of component selection, energy management, and performance of the vehicles at various speed conditions. The acceleration, deceleration, and braking are constant parameters to visualize the impact on the battery lifetime and performance due to the consecutive changes in the current profile of the battery. The rolling resistance, gravitational potential energy, aerodynamic drag, and vehicle inertia can be controlled by the positive tractive force of the vehicle. Based on the above statement, the commercial environment has been implemented with suitable drive cycles as per the road structure of countries^27,28^. The electrical product market is a challenging task for manufacturers due to the model of specimen validations should correlate with the standards of the particular country. According to the drive cycle pattern of the country, the electrical motor specifications may differ in power ratings and application of the EV. To continue the research on EV battery management, the vehicle specifications have been considered as shown in Table 3.

### Case 1. Performance analysis for Indian urban drive cycle

See Fig. 8.

**Fig. 8:**
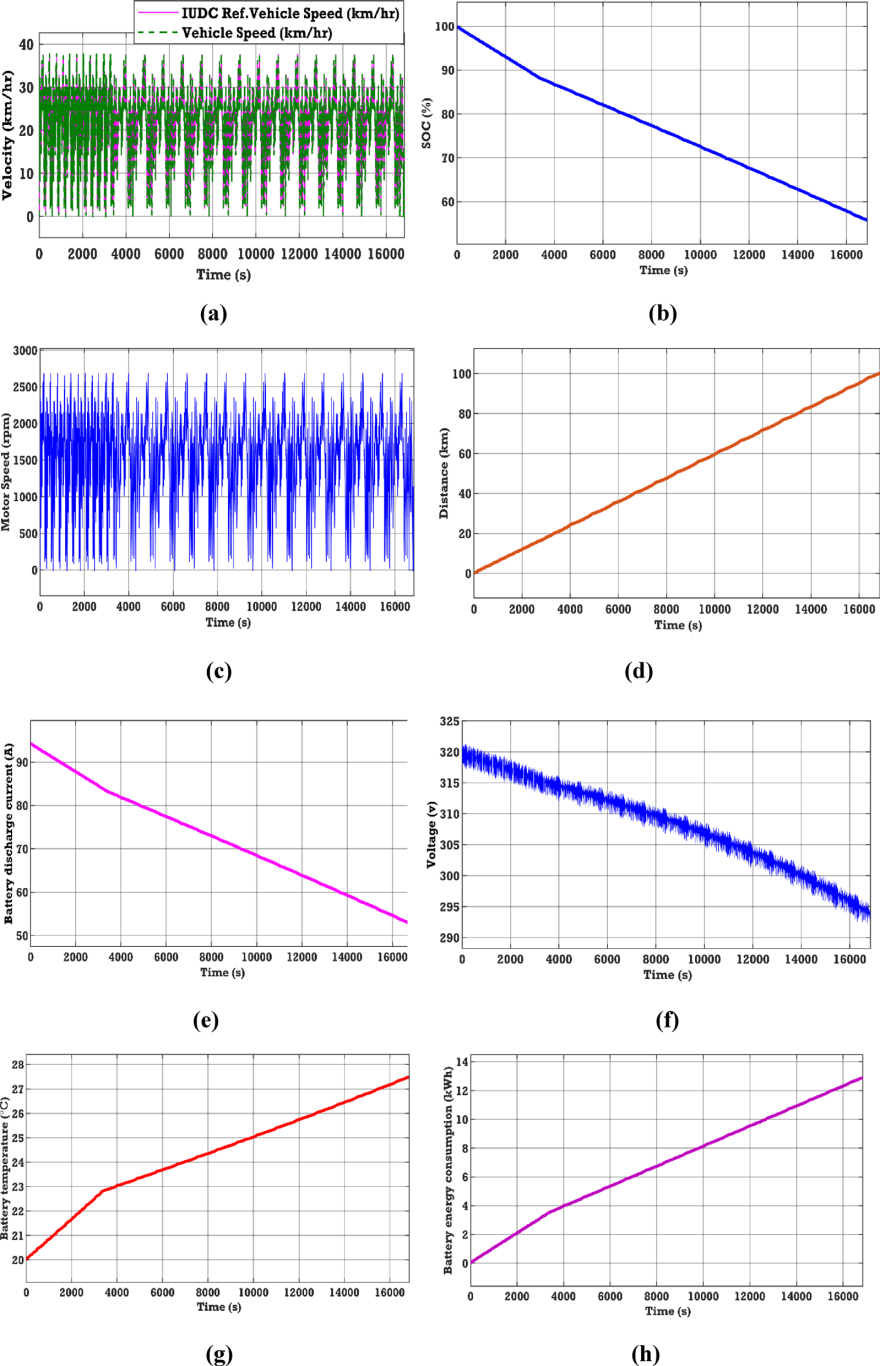
EV performance characteristics in urban drive cycle (**a**)–(**h**).

### Case 2. Performance analysis for the Indian highway drive cycle

See Fig. 9.

**Fig. 9:**
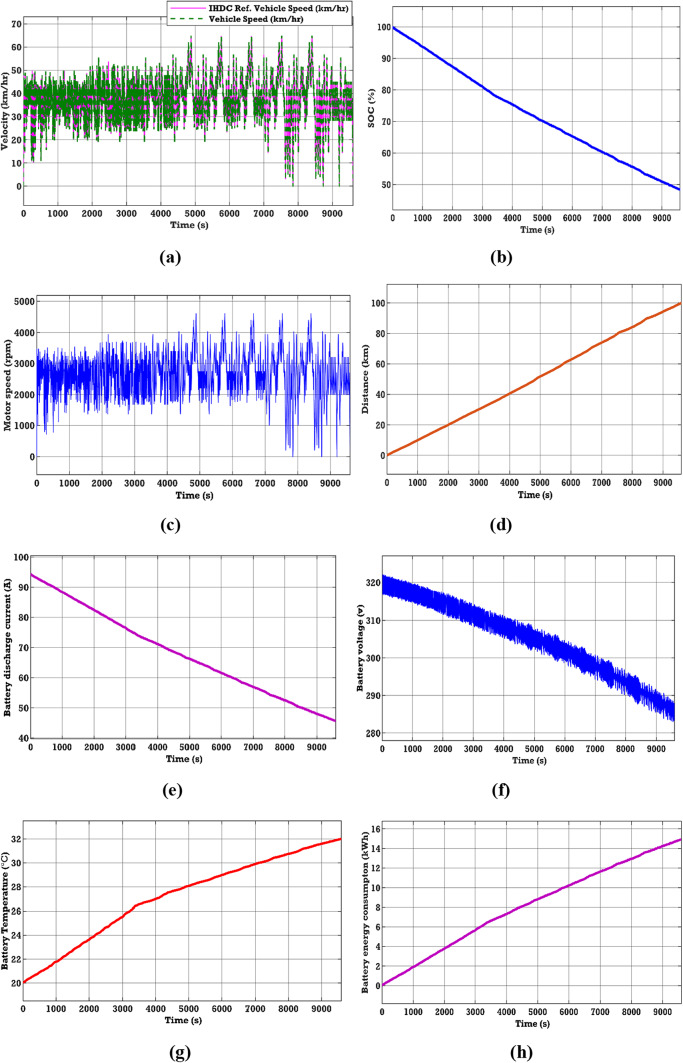
EV performance characteristics in the Highway drive cycle (**a**)–(**h**).

### Case 3. Performance analysis for modified Indian drive cycle

See Fig. 10.

**Fig. 10:**
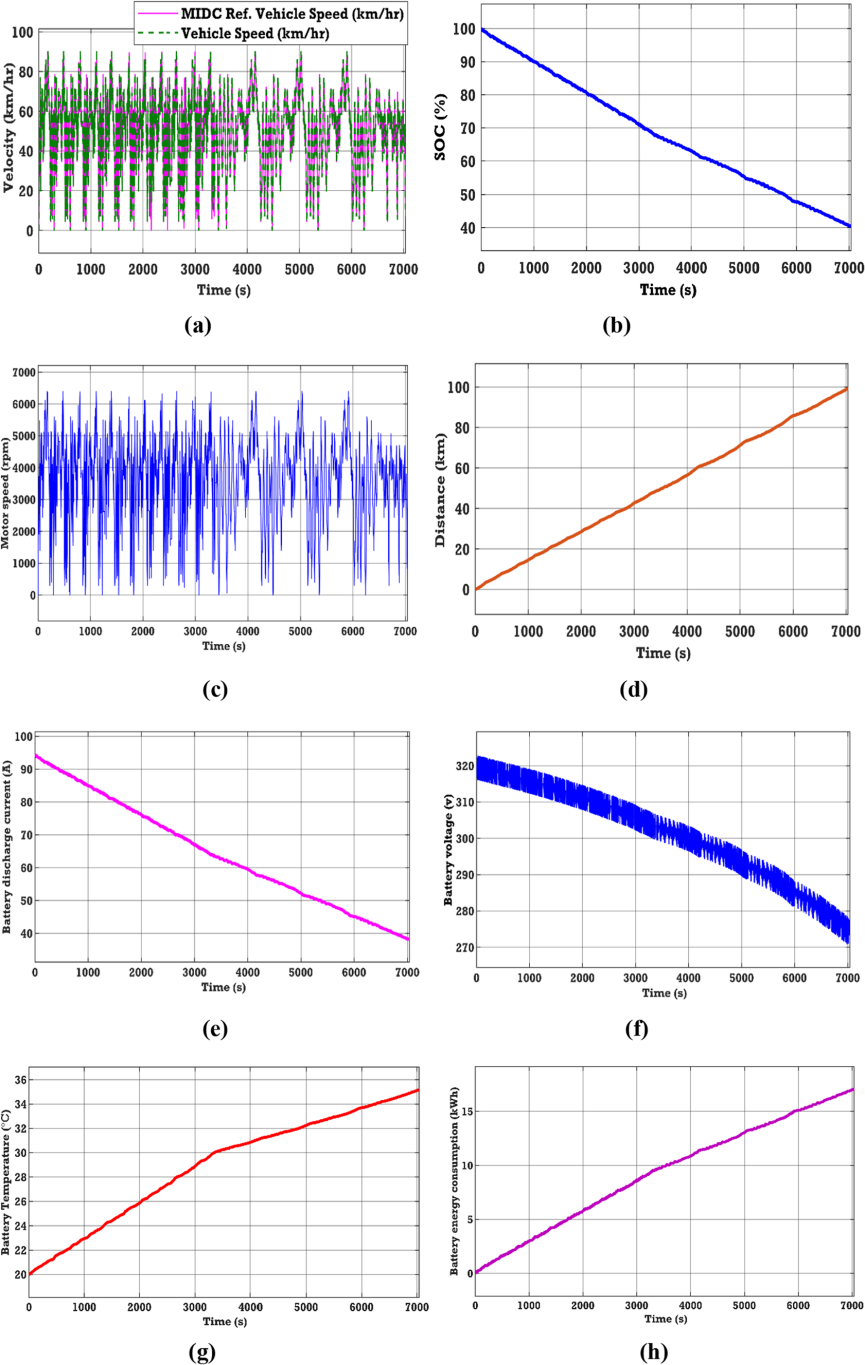
EV performance characteristics in Modified drive cycle (**a**)–(**h**) EV.

## The discussions of operational parameters and validations of the considered configuration of the EV power train

A virtual vehicle model was created in MATLAB/Simulink (version R2023 b), and the performance analysis has been implemented for a distance of 100 km of urban, highway, and modified pattern of drive cycle with the consideration of a 30.2 kW battery configuration. According to the distance, 15.73–21.84 kWh of energy has been consumed for all three Indian drive cycles. A comparative analysis of the performance of the EV battery throughout three distinct drive cycles is shown in Table 6.

**Table 6:** Comparison of EV battery performance on typical three distinct Indian drive cycles.

	IUDC	IHDC	MIDC
Maximum speed	37.66 (km/h)	64.74 (km/h)	90.07 (km/h)
Average speed	21.35 (km/h)	36.85 (km/h)	50.87 (km/h)
Motor speed	2688 (rpm)	4609 (rpm)	6405 (rpm)
Motor torque	85.70 (Nm)	95.31 (Nm)	98.11 (Nm)
SOC	55.79 (%)	48.46 (%)	40.54 (%)
Battery voltage	294.1 (v)	286.2 (V)	275 (V)
Battery current	9.37 (A)	13.28 (A)	16.16 (A)
Distance travelled	100 (km)	100 (km)	100 (km)
Battery discharge current	52.65 (A)	45.74 (A)	38.26 (A)
Battery temperature	27.5 (℃)	32.01 (℃)	35.15 (℃)
Battery energy consumption	0.129 (kWh/km)	0.149 (kWh/km)	0.171 (kWh/km)
Battery power used	0.69 (kW)	0.39 (kW)	0.5328 (kW)
Electrical efficiency	12.91 (kWh/100 km)	14.94 (kWh/100 km)	17.19 (kWh/100 km)

### Case 1

The EV battery’s performance has been assessed on the IUDC, characterized by a low mean speed and rapid acceleration. The IUDC attains a maximum velocity of 37 km/h and has a mean velocity of 21.2 km/h. From Fig. 8, the performance characteristics of various parameters of the EV battery in the IUDC source can be observed. Figure 8d shows the distance covered by the EV vehicle during the IUDC. In this case, a distance of 100 km has been considered for testing the drive system and evaluating the performance of the EV battery. Figure 8b and h show an energy consumption of 12.9 kWh and an SOC of 55.79% after traveling 100 km. In addition, from Fig. 8e–g, it can be observed that the battery discharge current of 52.65 (A), the voltage response of 294.1 (V), and the battery temperature increases from 20 to 27.5 °C.

### Case 2

The EV battery’s performance has been assessed on the IHDC, characterized by a low mean speed and rapid acceleration. The IHDC attains a maximum velocity of 37 km/h and has a mean velocity of 21.2 km/h. From Fig. 9, the performance characteristics of various parameters of the EV battery in the IUDC source can be observed. Figure 9d shows the distance covered by the EV vehicle during the IUDC. In this case, a distance of 100 km has been considered for testing the drive system and evaluating the performance of the EV battery. Figure 9b and h show an energy consumption of 14.94 kWh and an SOC of 48.46% after traveling 100 km. In addition, from Fig. 9e–g, it can be observed that the battery discharge current of 45.74 (A), the voltage response of 286.2 (V), and the battery temperature increment from 20 to 32.01 °C can be observed.

### Case 3

The EV battery’s performance has been assessed on the MIDC, characterized by a low mean speed and rapid acceleration. The MIDC attains a maximum velocity of 37 km/h and has a mean velocity of 21.2 km/h. From Fig. 10, the performance characteristics of various parameters of the EV battery in the IUDC source can be observed. Figure 10d shows the distance covered by the EV vehicle during the IUDC. In this case, a distance of 100 km has been considered for testing the drive system and evaluating the performance of the EV battery. Figure 10b and h show an energy consumption of 17.19 kWh and an SOC of 40.54% after traveling 100 km. In addition, from Fig. 10e–g, it can be observed that the battery discharge current is 38.26 (A), the voltage response is 275 (V), and the battery temperature increments from 20 to 35.15 °C.

This study demonstrates how the energy consumption and range of electric cars (EVs) in India are significantly impacted by various Indian driving cycles (IUDC, MIDC, and IHDC). By simulating EV performance under these driving cycles using a comprehensive MATLAB model, we have highlighted the influence of different driving styles on battery efficiency. The findings underscore the need for accurate range prediction algorithms that consider individual driving habits, as real-world driving patterns can lead to notable deviations from the legally authorized range.

The comparatively low maximum speed of 37.66 km/h and average speed of 21.35 km/h for the Indian Urban Driving Cycle (IUDC) suggest numerous stops and starts, which results in higher energy consumption because of continuous acceleration and deceleration. Although it is consistent with driving in cities, the motor’s lower speeds (2688 rpm) and higher torque (85.70 Nm) result in inefficiencies. The increased energy demand is reflected in the large decline in the state of charge (SOC) to 55.79%. Because of the frequent acceleration and deceleration, the battery voltage is 294.1 V, the current consumption is 9.37 A, and the discharge current is 52.65 A. Although the battery temperature is comparatively low at 27.5 °C, indicating less stress on the battery, the energy consumption is significant at 12.9 kWh, with an electrical efficiency of 12.91 kWh/100 km and a power utilization of 0.69 kW.

The greater maximum speed of 64.74 km/h and average speed of 36.85 km/h in the Indian Highway Driving Cycle (IHDC) indicate more stable driving circumstances, which are often more efficient for EVs. With a torque of 95.31 Nm, the motor speed is greater at 4609 rpm, indicating the increased requirement for maintaining higher speeds. Significant energy use is indicated by the SOC dropping to 48.46%. The discharge current is 45.74 A, the current draw is 13.28 A, and the battery voltage is 286.2 V. Because of the continuous increase in power output, the battery temperature is higher at 32.01 °C. The energy consumption is 14.91 kWh, with a power utilization of 0.39 kW and an electrical efficiency of 14.94 kWh/100 km.

The Modified Indian Driving Cycle (MIDC), which has an average speed of 50.87 km/h and a maximum speed of 90.07 km/h, simulates a combination of highway and urban driving situations. With a torque of 98.11 Nm, the motor reaches its maximum speed at 6405 rpm, indicating the need to sustain greater speeds. The cycle with the largest energy usage, as indicated by the SOC dropping to 40.54%. With a discharge current of 38.26 A and a draw of 16.16 A, the battery voltage is 275 V. With a maximum temperature of 35.15 °C, the battery is clearly under a lot of stress. With a power consumption of 0.5328 kW and an electrical efficiency of 17.19 kWh/100 km, the energy consumption is 17.1 kWh.

It is critical to recognise our model’s limits. This study did not take into account external elements that could impact EVs’ energy usage and range, including temperature and vehicle load. As demonstrated by the battery temperatures of 27.5 °C, 32.01 °C, and 35.15 °C for IUDC, IHDC, and MIDC, respectively, higher temperatures can raise battery temperature and affect efficiency. In a similar vein, changes in vehicle load can modify energy and power requirements. Future research should take these issues into account to improve the validity and dependability of our findings.

### Model limitations and external factors

Temperature: The Ambient temperature substantially influences the energy consumption and range of electric vehicles (EVs). Low temperatures might diminish battery efficiency and elevate energy consumption due to cabin warmth requirements. Conversely, elevated temperatures may result in heightened air conditioning usage, hence exacerbating energy consumption. Research indicates that the range of an electric vehicle can diminish by as much as 41% at − 7 °C in comparison to 24 °C. Subsequent research will integrate empirical temperature data to strengthen the reliability of our model.

Vehicle Load: The weight borne by an electric vehicle, encompassing passengers and cargo, directly affects energy usage. Increased loads elevate the energy used for acceleration and speed maintenance, thereby diminishing the overall range. For example, an increase in vehicle weight of 100 kg can reduce the range by around 5–10%. Our follow up research will involve varied vehicle loads to provide a more full knowledge of their influence.

While our findings are based on simulations employing Indian driving cycles, the results may not be generalizable to other situations or regions. Factors such as road conditions, traffic patterns, and driving behaviors might differ greatly. Future studies will involve validating our model using experimental data from varied driving scenarios to increase its applicability and dependability.

## Conclusion

This study shows how the energy consumption and range of electric cars (EVs) in India are significantly impacted by the various Indian driving cycles (IUDC, MIDC, and IHDC). We have shed light on how different driving styles impact battery efficiency by simulating EV performance under these various driving cycles using a comprehensive MATLAB model. The findings highlight the need for accurate range prediction algorithms that consider individual driving habits by demonstrating that real-world driving patterns can result in notable deviations from the legally authorized range.

According to the study, the Modified Indian Driving Cycle (MIDC) uses energy more evenly than the Indian Urban Driving Cycle (IUDC), which consumes more energy due to frequent stops and starts. Specifically, the energy consumption for IUDC is 12.9 kWh/100 km, while for MIDC, it is 17.19 kWh/100 km. Furthermore, determining the ideal average speed range that uses the least amount of battery energy offers useful advice for enhancing range efficiency for both EV producers and consumers. For example, the average speed for IUDC is 21.35 km/h, while for MIDC, it is 50.87 km/h.

Enhancing driving behaviors may be a crucial strategy for increasing the useful range of electric vehicles in India, according to these studies, highlighting the significance of customized drive cycles in the certification process. The research’s practical significance stems from its ability to guide the design and optimization of electric vehicles for Indian driving conditions, which would eventually improve user satisfaction, lower emissions, and increase energy efficiency.

In subsequent studies, we intend to use real experimental data to verify the model’s predictions and take into account the impact of outside variables like weather and vehicle load on energy use. This will improve the’ robustness and dependability and offer a more thorough comprehension of how well electric vehicle batteries operate under different driving circumstances.

## Data Availability

The datasets used and/or analysed during the current study available from the corresponding author on reasonable request. The confidentiality of this research necessitates the restriction of some raw data. However, the minimal dataset necessary to replicate the findings presented in this manuscript is provided.

## Abbreviations

IDC: Indian drive cycle
IUDC: Indian urban driving cycle
IHDC: Indian highway driving cycle
MIDC: Modified Indian driving cycle
WLTP: World harmonized light vehicles testing protocol
SOC: State of charge
IC: Internal combustion
SUV: Sport utility vehicle
BEV: Battery electric vehicle
EV: Electric vehicle
FoC: Field-oriented control
PMSM: Permanent magnet synchronous motor
NEDC: New European driving cycle

## List of symbols

$${\text{F}}_{{\text{T}}}$$: Tractive force (N)
${\text{F}}_{{\text{L}}}$: Forces exerted on the vehicle (N)
${\text{v}}_{{\text{t}}}$: Velocity of the vehicle (km/h)
$$F_{gt}$$: Gravitational force (N)
${\text{F}}_{{{\text{Roll}}}}$: Rolling resistance force (N)
$${\text{F}}_{{{\text{AeoD}}}}$$: Aerodynamic drag (N)
${\text{R}}_{{{\text{c}}0}} ,{\text{R}}_{{{\text{c}}1}}$: Coefficients of rolling resistance
$\theta$: Gradient angle (degrees)
${\text{D}}_{{\text{c}}}$: Coefficient of drag
ρ: Air density (kg/m^3^)
${\text{v}}_{0}$: Headwind velocity (km/h)
${\text{r}}_{{\text{d}}}$: Gear reduction ratio
$M_{eff}$: Effective vehicle mass (kg)
${\text{J}}_{{\text{t}}}$: Tire inertia (kg m^2^)
${\text{J}}_{{{\text{mi}}}}$: Motor inertia-mass (kg m^2^)
${\text{I}}_{{\text{C}}}$: Charging current of the battery (A)
$V_{n}$: Battery voltage at nominal (V)
$\eta_{Q}$: Total charge performance
${\upeta }_{{\text{D}}}$: Total discharge performance
$I_{D}$: Battery discharge current (A)
$T_{t}$: Tire torque (Nm)
$$t_{R}$$: Tire radius (m)
$t_{w}$: Tire speed (km/h)
$P_{out, m}$: Motor output power (kW)
$B_{Ah}$: Battery capacity (Ah)
$T_{m}$: Motor torque (Nm)
$$\eta_{T}$$: Transmission efficiency
$W_{m}$: Speed of the motor (rpm)
$B_{P}$: Battery power (kWh)
$$P_{eff}$$: Motor efficiency
$$inv_{eff}$$: Inverter efficiency
$$V_{Dc}$$: Battery voltage (V)
$$r$$: Translatory speed (m/s)
$w$: Angular speed (rad/s)
$H$: The vertical load exerted on the tire
S: Tire slip
$\varepsilon$: Tractive effort coefficient
